# Supplementary material for: The genome sequence of the giant tachinid fly, Tachina grossa (Linnaeus, 1758)
Source: Wellcome Open Res. 2024 Oct 7;9:571. [Version 1] doi: 10.12688/wellcomeopenres.23102.1 (PMC11602700; doi:10.12688/wellcomeopenres.23102.1)
Supplement: Peer review history

# Reviewer report: Reviewer response for version 1

**DOI:** 10.21956/wellcomeopenres.25439.r111277

**Published:** 2024-11-27

**Authors:** Bhagya C.Thimmappa

**Affiliations:** 1 ARS, US Department of Agriculture, Chatsworth, NJ, USA

In the work titled “The genome sequence of the giant tachinid fly, *Tachina grossa *(Linnaeus, 1758), authors report the first nuclear and mitochondrial genome assembly for female *Tachina grossa *along with nuclear genome annotation. The nuclear genome is assembled to chromosome level with a length of 936.90MB, and it is nearly complete with a busco value of 98.4% based on the diptera_odb10 dataset.

 The assembly and annotation reported here are valuable for future evolutionary and comparative research on Tachinids. The authors provide enough introduction, and the manuscript reads well with a well-documented method section.

**Minor comments:**

1. Is there a reason for sequencing female *Tachina grossa,* not male?
2. How can one distinguish male *Tachina grossa *from female?

Are sufficient details of methods and materials provided to allow replication by others?

Yes

Is the rationale for creating the dataset(s) clearly described?

Yes

Are the datasets clearly presented in a useable and accessible format?

Yes

Are the protocols appropriate and is the work technically sound?

Yes

Reviewer Expertise:

Bioinformatics, genomics, multiomics

I confirm that I have read this submission and believe that I have an appropriate level of expertise to confirm that it is of an acceptable scientific standard.

# Reviewer report: Reviewer response for version 1

**DOI:** 10.21956/wellcomeopenres.25439.r104686

**Published:** 2024-10-21

**Authors:** Marcus C Stensmyr

**Affiliations:** 1 Lund University, Lund, Sweden

A chromosome length assembly of the giant tachinid fly is here presented. The genome is part of the Tree of Life (ToL)  effort, and the genome has been sequenced, assembled and annotated using the ToL pipeline. The genome (936.9MB)  is assembled in 6 chromosomal pseudomolecules, plus the mitochondria. The giant tachinid fly is an interesting species, and a Tachinidae genome assembly a welcome addition to Diptera genomic research.

Are sufficient details of methods and materials provided to allow replication by others?

Yes

Is the rationale for creating the dataset(s) clearly described?

Yes

Are the datasets clearly presented in a useable and accessible format?

Yes

Are the protocols appropriate and is the work technically sound?

Yes

Reviewer Expertise:

Diptera genomics, neurogenetics

I confirm that I have read this submission and believe that I have an appropriate level of expertise to confirm that it is of an acceptable scientific standard.
